# An Improved Method of Brain-Localization in Epilepsy

**Published:** 1894-03

**Authors:** 


### EDITORIAL DEPARTMENT.

F. E. DANIEL, M. D., Editor.
S. E. HUDSON, M. D., Managing Editor.
A. J. SMITH. M. D., Galveston, Associate Editor.

EDITORIAL STAFF:

PROF. J. E. THOMPSON, M. D., Texas Medical College, Galveston; Surgery.
PROF. WM. KEILLER, M. D., Texas Medical College, Galveston; Obstetrics and Gynecology.
PROF. DAVID CERNA, M. D., Texas Medical College, Galveston; Therapeutics.
PROF. A. J. SMITH, M. D., Texas Medical College, Galveston; Medicine
DR. ISADORE DYER, Tulane University; Dermatology.
DR. R. H. L. BIBB, Saltillo, Mexico; Foreign Correspondent.
DR. ROBT. MORRIS, Charity Hospital, N. O.; Clinical Reports

### "AN IMPROVED METHOD OF BRAIN LOCALIZATION IN EPILEPSY."

We have read with much interest, a very valuable paper under the above title, by Dr. B. E. Hadra, of Galveston, contributed to the Southern Surgical and Gynecological Association, at its recent New Orleans meeting, and published in the last February number of the *Annals of Surgery.* The idea inculcated by Dr. Hadra, though still in an embryonic condition, certainly merits further and serious consideration.

It must be conceded that the surgical treatment of epilepsy, whether the disease be idiopathic or traumatic, is not, after all, acompanied with so much failure or actual evil as might be supposed by a careless or biased observer, provided the operation bears upon its very face *its raison d'etre.* The measure has succeeded in many cases in which prolonged and varied internal medication had proved of no avail whatever. The method, however, is more likely to fail with those operators ever eager to use the knife at all hazards, without a proper previous study of the case before them, or with those others, again, who, thoroughly convinced of the justifiability of the operation merely from the history and symptomatology of the case, yet work in the dark, without a sufficient knowledge of the physiology of cerebral localization.

Trephining for undoubted traumatic, and even for idiopathic epilepsy, is of acknowledged value, but the abuse of it, especially in idiopathic cases, is apt to lead to much and perhaps irreparable evil.

We do not deny, then, the value of trephining in epilepsy, and statistics show that the procedure has produced most excellent and even permanent results. Thus, of the thirty cases recorded by Briggs, twenty-five were cured, three were relieved, one received no benefit, and one died, probably from the effects of the operation. According to Walsham, out of 130 cases, seventy-five were entirely cured, eighteen improved, and thirty died. These statistics alone give a per centage of 62½ of recoveries, a result more brilliant than the best furnished so far by any other form of medication. Later reports have confirmed and continue to confirm almost daily, the value of the surgical measure under consideration. "The necessity for operation becomes very evident," says a good authority, "if we can rely on the assertion of Garmany that fifty per cent. of all cases of frontal traumatism become epileptic." Yet, the young and inexperienced practitioner, as well as his elder brother whom he tries to imitate, bold, it is true, but often painfully ignorant of even the rudiments of brain localization, are both most too ready to jump at surgical conclusions, suiting the action to the thought. Some surgeons, indeed, go so far as to trephine in any case of obstinate epilepsy in the hope of directly removing the diseased portion of the brain substance that is supposed to be causing the trouble. Now, is this always justifiable? We mean, of course, no disparagement to the friends of the knife, but modern surgery, bold though it may be, should not lose sight of the fact that the operation of trephining by itself, "is too severe to be performed in the dark." Lucas-Championniére has but recently remarked that it is an error to suppose that in Jacksonian epilepsy operation is always indicated, or that it always affords a clear guide to the point to be attacked; and in support of his statement refers to four instances occurring in his own practice. He agrees with Horsly in that trephining is a serious procedure, when there are extensive lesions, and that it is best to open the cranium freely, rather than to uncover a spot determined in advance, in the hope of coming upon a strictly localized lesion.

If we were to summarize the classes of indications for trephining, we would mention the following as the chief ones, as recently set forth by Broca, that is: "1. We may be guided by external lesions, as in the case of traumatism. 2. We may seek for a cerebral lesion, of known or unknown nature, the situation of which is diagnosed by the symptoms induced, in the light of our knowledge of the functions of different portions of the brain. 3. We may operate without being so guided, when the nature of the lesion has been determined, from experience as to the usual seat of such lesions, and of the best way to approach them." But it must not be forgotten (and we make this frank statement conscientiously) that our knowledge of the focal centres is as yet imperfect, a subject which urgently demands further investigation. Epilepsy *per se*, as far as our knowledge goes, is not a disease, but a symptom, and here precisely lie the difficulties, at least in a large number of cases, of pointing out the lesion which causes the trouble. To this end several methods have been suggested, but not one of them has given, so far, complete satisfaction.

In a given case, after we have exposed the brain, how are we to determine the seat of the lesion? This is the question, that as yet, remains unanswered satisfactorily, if we except perhaps Dr. Hadra's suggestion.

After pointing out some of the sources of the difficulties, mistakes and errors in the present methods, such as lacking opportunity to witness the initial signal; erroneous statements of the patients or their relatives; the great number of brain foci, which must exist, but which are not yet discovered; the fact that the brain foci are not, so far as we know, strictly defined localities; that the topographical, as well as the electrical localization, does not designate the seat of the morbid process, but that both only map out the physiological focus belonging to a certain group of muscles; after confessing, in short, that the present methods of detecting the real seat of the disease are not sufficient, unless the pathological changes are plainly seen or felt, since as soon as the surgeon has to depend upon topographical or electrical localization alone, he may be misled because he is only striving to map out the physiological focus of the muscles that have given the initial signal, Dr. Hadra concludes that there are no positive proofs that this focus, when so determined, really is the diseased portion of the cortex. He goes on further to say, and properly we believe, that such methods are no guides as to the extent of the disease, and finally, proposes the following method: "*My advice is*," he says, "*to use the induced current not solely to find the physiological focus belonging to the muscles giving the initial signal, but to find the spot from where an epileptic attack of the same nature the patient is accustomed to suffer from*,

*can be started, independently of the fact whether those two points coincide or not.*" With this rule, Dr. Hadra believes that it is not necessary at all to be so scrupulous in localizing the part attacked before the operation, although there is no objection to this. He asserts that his method has given full satisfaction in two cases, cases which he details in his interesting paper.

It may be argued that when a strong current of electricity is applied to the brain, the irritation produced on the healthy cortex may be sufficient by itself to cause epileptic phenomena. This alone would be an objection to the method proposed by Dr. Hadra, and apt to mislead. Convulsive movements, however, of a certain group of muscles, caused by electrical stimulation of healthy brain substance, are not epileptiform, and it is only after a prolonged and repeated electrization, according to Ferrier, that a monkey may be rendered epileptic. It follows, then, that practically speaking electrical stimulation of a healthy cortex, is not necessarily followed by epileptic symptoms. Yet, when the diseased portion of the brain substance has been rendered morbidly epileptic, no matter by what influences, the specific energy of the cells involved, under such circumstances, can then be aroused by any kind of stimulus. In these instances, of course, no more decided diagnosticating element can be employed than electricity. In phsyiological conditions, it is often difficult to produce peripheral effects, even by very strong currents applied to the brain, and yet over an epileptic cortex, especially when the precise spot is found, the slightest touch by the electrodes will cause a fit of epilepsy.

This sort of argumentation, logical to say the least, and coupled with experimental evidence, though insufficient, leads Dr. Hadra to entertain hopes for the continued success of his method, and, frankly, we do not see why this should not be tried still further. Regarding another objection that might arise in the minds of many, with reference to injurious effects caused by the use of the current as proposed, there appears to be no foundation for such objection, and Dr. Hadra makes the assertion to the effect that, in his experience, "prolonged use of the galvanic current, even of a strength of 12 to 20 milliampéres, and repeated daily for many months, had not only no unwholesome, but on the contrary, a very beneficial effect upon the functions of the brain;" that there is, therefore, no excuse for not trying to get reliable information regarding the true location of the diseased portion of the brain in epileptic subjects, "by the use of extensive and, if necessary, repeated application of the induced current,'' without even fearing to bring about upon the patient, while on the table, two or more attacks ot the symptom.

We cannot foretell to any degree of certainty the ultimate fate of Dr. Hadra's method, but if this, by further trial (which it certainly deserves), should prove to be of superior advantage as is apparently claimed for it, the author shall have rendered an important and lasting service to brain surgery, and particularly to suffering humanity.

But, *a propos* of this same subject, it seems to us that generally too much stress is laid upon the motor areas of the cortex as the location of irritation in epilepsy. The irritation, on the other hand, can be traced more often than is generally supposed to the sensory regions, as in cases which present unilateral hallucinations, so to speak, ''confined in origin,'' as some one has properly remarked, ''to the limited auditory space, on the right side or the left.'' It is evident, therefore, that in these instances the irritation has been primarily located in the *sensory* centres, that is, over the first and second temporal convolutions, from which it has passed to the motor regions, and there caused the corresponding discharge. Does it not follow from this, that in these cases it is the sensory and *not* the motor centres, the former the true origin of the irritation, that should be the objective point to be considered by the surgeon? This is mind, and properly carried into execution, there is no reason why fewer failures should not be recorded in the surgical treatment of epilepsy. This suggestion is not original with us, but we mention it simply to emphasize the importance of always bearing in mind a point that is generally neglected by the thoughtlesss and over-enthusiastic surgeon. D. C.

